# Changing Healthcare Professionals’ Attitudes about Dementia with Lived Experience Storytelling

**DOI:** 10.1093/geroni/igaf122.166

**Published:** 2025-12-31

**Authors:** Edward Cisek, Olivia Cohen, Anne Kenny, Amy Puca, Vicki Zimmer, Stephani Shivers

**Affiliations:** CaringKind, New York, New York, United States; CaringKind, New York, New York, United States; CaringKind, New York, New York, United States; Via Evaluation, Buffalo, New York, United States; Via Evaluation, Buffalo, New York, United States; CaringKind, New York, New York, United States

## Abstract

People living with dementia (PLWD) can combat stigma by sharing their insights and experiences and giving feedback on services they receive. Via an Administrative on Community Living – Alzheimer’s Disease Program Initiatives grant, CaringKind, a dementia-focused community-based organization, launched Dementia Act II (DA2), a program aiming to change attitudes and behaviors of healthcare, research, and industry professionals/students towards PLWD. Four facilitated groups of PLWD crafted scripts about their healthcare experiences over 4-6 sessions and presented in a one-hour grand rounds format. Before and after the presentations, audiences were asked to write three words describing the experience of a PLWD. Post-performance, participants were also asked to respond on a 5-point scale from 0 (“never”) to 4 (“every time”) regarding their past/anticipated future interactions with PLWD around 3 themes. Results revealed the most common descriptive words changed from “frustration”, “memory”, “confusion” and “sad” to “hope”, “resilient”, “creative” and “strong” (n = 84). Furthermore, compared to their prior interactions, participants are significantly more likely to speak directly to the PLWD and with empathy and candor (t(62)=-6.43, p<.001); consider the PLWD’s involvement in peer support and meaningful engagement (t(63)=-9.60, p<.001); and consider the connectivity of those family/friends providing care to ongoing support, education, and planning services as care needs change (t(64)=-10.45, p<.001). We conclude that by hearing about PLWD experiences in their own words, practicing professionals improve their impression of PLWD and may modify how they interact with, and plan for the support and care of, families affected by dementia.

